# Caloric Restriction Mimetic 2-Deoxyglucose Alleviated Inflammatory Lung Injury *via* Suppressing Nuclear Pyruvate Kinase M2–Signal Transducer and Activator of Transcription 3 Pathway

**DOI:** 10.3389/fimmu.2018.00426

**Published:** 2018-03-02

**Authors:** Kai Hu, Yongqiang Yang, Ling Lin, Qing Ai, Jie Dai, Kerui Fan, Pu Ge, Rong Jiang, Jingyuan Wan, Li Zhang

**Affiliations:** ^1^Department of Pathophysiology, Chongqing Medical University, Chongqing, China; ^2^Department of Physiology, Chongqing Medical University, Chongqing, China; ^3^Hospital of Chongqing University of Arts and Sciences, Chongqing, China; ^4^Laboratory of Stem Cell and Tissue Engineering, Chongqing Medical University, Chongqing, China; ^5^Department of Pharmacology, Chongqing Medical University, Chongqing, China

**Keywords:** caloric restriction mimetic, 2-deoxyglucose, pyruvate kinase M2, signal transducer and activator of transcription 3, inflammation

## Abstract

Inflammation is an energy-intensive process, and caloric restriction (CR) could provide anti-inflammatory benefits. CR mimetics (CRM), such as the glycolytic inhibitor 2-deoxyglucose (2-DG), mimic the beneficial effects of CR without inducing CR-related physiologic disturbance. This study investigated the potential anti-inflammatory benefits of 2-DG and the underlying mechanisms in mice with lipopolysaccharide (LPS)-induced lethal endotoxemia. The results indicated that pretreatment with 2-DG suppressed LPS-induced elevation of tumor necrosis factor alpha and interleukin 6. It also suppressed the upregulation of myeloperoxidase, attenuated Evans blue leakage, alleviated histological abnormalities in the lung, and improved the survival of LPS-challenged mice. Treatment with 2-DG had no obvious effects on the total level of pyruvate kinase M2 (PKM2), but it significantly suppressed LPS-induced elevation of PKM2 in the nuclei. Prevention of PKM2 nuclear accumulation by ML265 mimicked the anti-inflammatory benefits of 2-DG. In addition, treatment with 2-DG or ML265 suppressed the phosphorylation of nuclear signal transducer and activator of transcription 3 (STAT3). Inhibition of STAT3 by stattic suppressed LPS-induced inflammatory injury. Interestingly, posttreatment with 2-DG at the early stage post-LPS challenge also improved the survival of the experimental animals. This study found that treatment with 2-DG, a representative CRM, provided anti-inflammatory benefits in lethal inflammation. The underlying mechanisms included suppressed nuclear PKM2-STAT3 pathway. These data suggest that 2-DG might have potential value in the early intervention of lethal inflammation.

## Introduction

Uncontrolled inflammation is one of the primary mechanisms responsible for the lethal outcomes in patients with critical illness (1, 2). To restrict inflammatory injury, the inflammation-related signaling pathways have been extensively studied, and various targets have been proposed for anti-inflammatory therapy (3, 4). In addition, recent studies indicate that inflammation is an energy-intensive process with significant metabolic reprogramming (5), which implies that modulation of immunometabolism might become a novel strategy to limit inflammatory injury (6).

Caloric restriction (CR), also known as dietary restriction, is a natural intervention to restrict energy metabolism (7). The increasing evidence suggest that CR is a reliable approach to prolong life span and increase health span in both experimental animals and human beings (8, 9). However, CR also disturbs some physiological functions, which limit its practicability in patients (10). Therefore, CR mimetics (CRM) have been developed to mimic the beneficial effects of CR without reducing food intake (11). Because glucose is the primary energy source, the glycolytic inhibitor 2-deoxyglucose (2-DG) was proposed as a representative CRM (12). Several studies indicate that the beneficial effects of CR and CRM might be associated with suppressed inflammatory response (13, 14), but the underlying mechanisms remains unclear.

Recently, the pivotal roles of pyruvate kinase M2 (PKM2), an unique isoform of the pyruvate kinases, in inflammatory response have been revealed (15). PKM2 locates in both cytoplasm and nucleus. The cytoplasmic PKM2 functions as a metabolic kinase that catalyzes the final rate-limiting step of glycolysis (16). On the contrary, the nuclear PKM2 functions as a protein kinase that phosphorylates its downstream targets and facilitates transcriptional activation (17). Inflammatory stimuli could suppress the metabolic activity of PKM2 but induce the nuclear accumulation of PKM2, which promotes the expression of pro-inflammatory mediators (18). Thus, the nuclear PKM2 provides a molecular mechanism to bridge metabolism and inflammation (19).

In this study, the potential anti-inflammatory benefits of 2-DG were investigated in mice exposed to lethal dose of lipopolysaccharide (LPS), a major pro-inflammatory stimulus originated from Gram-negative bacterial (20). In addition, the underlying mechanisms were investigated *via* determination of the modulatory effects of 2-DG on nuclear PKM2 and the phosphorylation status of its downstream target, such as signal transducer and activator of transcription 3 (STAT3) (17).

## Materials and Methods

### Materials

Lipopolysaccharide (from *Escherichia coli*, 055:B5) and 2-DG were the products of Sigma (St. Louis, MO, USA). The PKM2 activator ML265, the STAT3 inhibitor stattic, and the myeloperoxidase (MPO) activity assay kit were the products of Cayman Chemical (Ann Arbor, MI, USA). The enzyme-linked immunosorbent assay (ELISA) kits for the determination of mouse tumor necrosis factor alpha (TNF-α) and interleukin 6 (IL-6) were the products of NeoBioscience Technology Company (Shenzhen, China). The nuclear protein extract kit was the product of Genechem Co., Ltd. (Shanghai, China). The rabbit anti-mouse PKM2 (D78A4), STAT3 (79D7), phospho-STAT3 (D3A7), and lamin B (D9V6H) antibodies were the products of Cell Signaling Technology (Danvers, MA, USA). The horseradish peroxidase-conjugated goat anti-rabbit antibody was the product of Proteintech (Wuhan, China). The BCA protein assay kit was the product of Thermoscientific (Rockford, IL, USA). The enhanced chemiluminescence (ECL) reagents were the products of Advansta (Menlo Park, CA, USA).

### Animals

Male BALB/c mice, 6 to 8 weeks old, were provided by the Experimental Animal Center of Chongqing Medical University. The mice were housed in a specific pathogen-free room under a 12 h dark/light cycle (temperature: 20–25°C, relative humidity: 50 ± 5%) and acclimatized for 1 week before use. The mice were fed with a standard laboratory diet and water *ad libitum*. The experimental procedures were approved by the Animal Care and Use Committee of Chongqing Medical University.

### Lethal Endotoxemia

Lethal endotoxemia was induced in mice with intraperitoneal injection of LPS (20 mg/kg). The mice were killed 18 h after LPS challenge, and the plasma and lung samples were harvested. To evaluate the potential effects of 2-DG, the mice were treated with 2-DG (500 mg/kg, dissolved in normal saline, i.p.) 30 min before LPS exposure. To investigate the underlying mechanism of 2-DG, the mice were co-treated with the PKM2 activator ML265 (50 mg/kg, dissolved in DMSO, i.p.) or STAT3 inhibitor stattic (5 mg/kg, dissolved in DMSO). To determine the mortality rate, survival of the mice (*n* = 18 per group) was assessed every 6 h for at least 7 days, and the cumulative survival rate was depicted using the Kaplan–Meier curve.

### Histological Analysis

The lung samples were fixed in formalin, followed by routine dehydration. Then the samples were embedded in paraffin, and 4-µm sections were prepared. Standard hematoxylin and eosin staining were performed for histopathological analysis under a light microscope (Olympus, Tokyo, Japan). The histopathological abnormalities of the lung sections were blindly scored based on the method described previously with slight modifications (21). Briefly, the histological alterations were graded on a scale of 0–4 (0, normal; 1, light; 2, moderate; 3, strong; 4, intense) for the following pathological features: congestion, edema, inflammation, and hemorrhage. A cumulative total histology score for all of the parameters was calculated.

### Determination of Evans Blue Leakage

Evans blue dye (80 mg/kg) was intravenously injected into the mice 1 h before the termination of the experiment to determine the degree of pulmonary vascular leakage as previously reported with slight modifications (22). At the end of the experiment, the lungs were perfused free of blood with PBS (containing 5 mM EDTA) *via* thoracotomy. The lungs were excised, blotted dry, and homogenized in PBS. The homogenates were incubated with two volumes of formamide at 60°C for 24 h and then centrifuged at 200 *g* for 10 min. The supernatants were harvested, and the optical density was determined spectrophotometrically at 620 nm. The concentration of Evans blue was calculated according to the standard curve.

### Determination of MPO Activity

The enzyme activities of MPO were determined by the MPO assay kit according to the manufacturer’s instructions (Cayman Chemical, USA). Briefly, the frozen lung samples were homogenized in a cell-based assay buffer. Then the homogenates were centrifuged at 200 *g* for 10 min at room temperature. The supernatants were mixed with the assay buffer and incubated with the MPO substrate 3,3′5,5′-tetramethyl-benzidine (TMB) at 37°C for 5 min. The chemical reaction yields a blue color detectable by its absorbance at 650 nm. The MPO activities were calculated according to the standard curve and normalized by the protein concentration of each sample.

### Detection of Pro-inflammatory Cytokines by ELISA

The plasma and pulmonary levels of TNF-α and IL-6 were determined using the ELISA kits according to the manufacturer’s instructions (NeoBioscience). The concentration of TNF-α or IL-6 was calculated according to the standard curve. The levels of TNF-α and IL-6 in lung homogenates were normalized by the protein concentration of each sample.

### Western Blot Analysis

The total proteins or nuclear proteins were prepared, and the protein extracts were fractionated on polyacrylamide–SDS gel and then transferred to nitrocellulose membrane. The membrane was blocked with 5% (w/v) non-fat milk in Tris-buffered saline containing 0.05% Tween-20, and then the membrane was incubated with the primary antibodies against PKM2, phospho-STAT3, STAT3, or lamin B overnight at 4°C. After washing, the membrane was incubated with secondary antibody. Antibody binding was visualized with ECL reagents and the ChemiDoc Touch Imaging System (Bio-Rad).

### Statistical Analysis

The experimental data were expressed as mean ± SD. The statistical significance among means was analyzed by one-way ANOVA, followed by the Turkey’s *post hoc* test. In addition, the Kaplan–Meier curve and log-rank test were performed for survival analysis. Results were considered statistically significant when *P* < 0.05.

## Results

### 2-DG Alleviated LPS-Induced Lethal Inflammation

**Figure 1:**
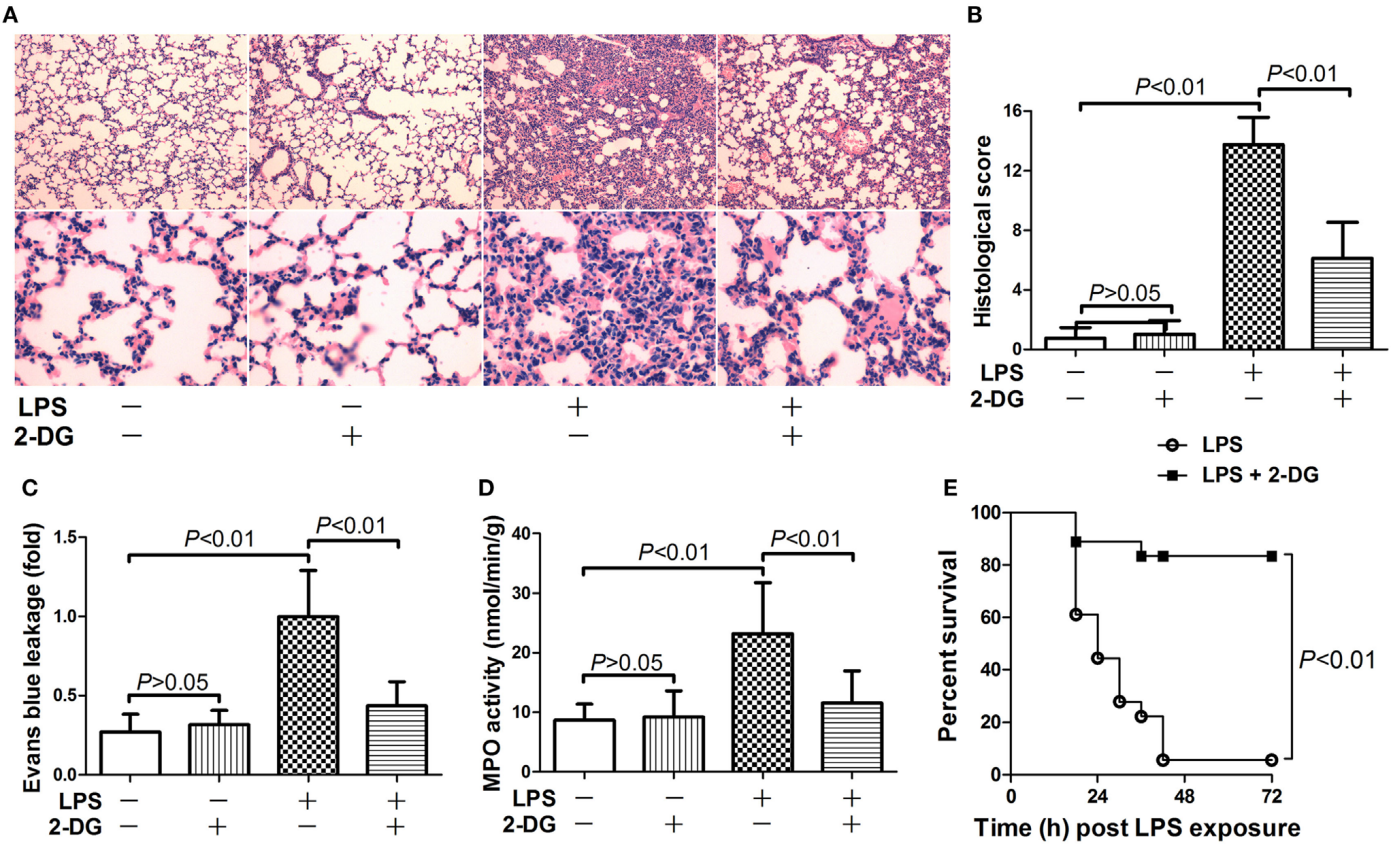
2-Deoxyglucose (2-DG) attenuated lipopolysaccharide (LPS)-induced lung injury and mortality. **(A)** Mice with LPS-induced lethal inflammation were treated with vehicle or 2-DG 30 min before LPS exposure. **(A)** The lung samples were harvested 18 h after LPS exposure, and the lung sections were stained with hematoxylin and eosin for morphological evaluation. The representative lung sections of each group were shown. **(B)** The histopathological abnormalities of the lung sections were blindly scored. **(C)** The degree of Evans blue leakage was determined. **(D)** The myeloperoxidase (MPO) activities in lung tissue were determined. Data were expressed as mean ± SD, *n* = 8. **(E)** Another set of animals were exposed to LPS and treated with vehicle or 2-DG, the mortality of the animals was monitored, and the percent survival rate was expressed as a Kaplan–Meier survival curves, *n* = 18.

**Figure 2:**
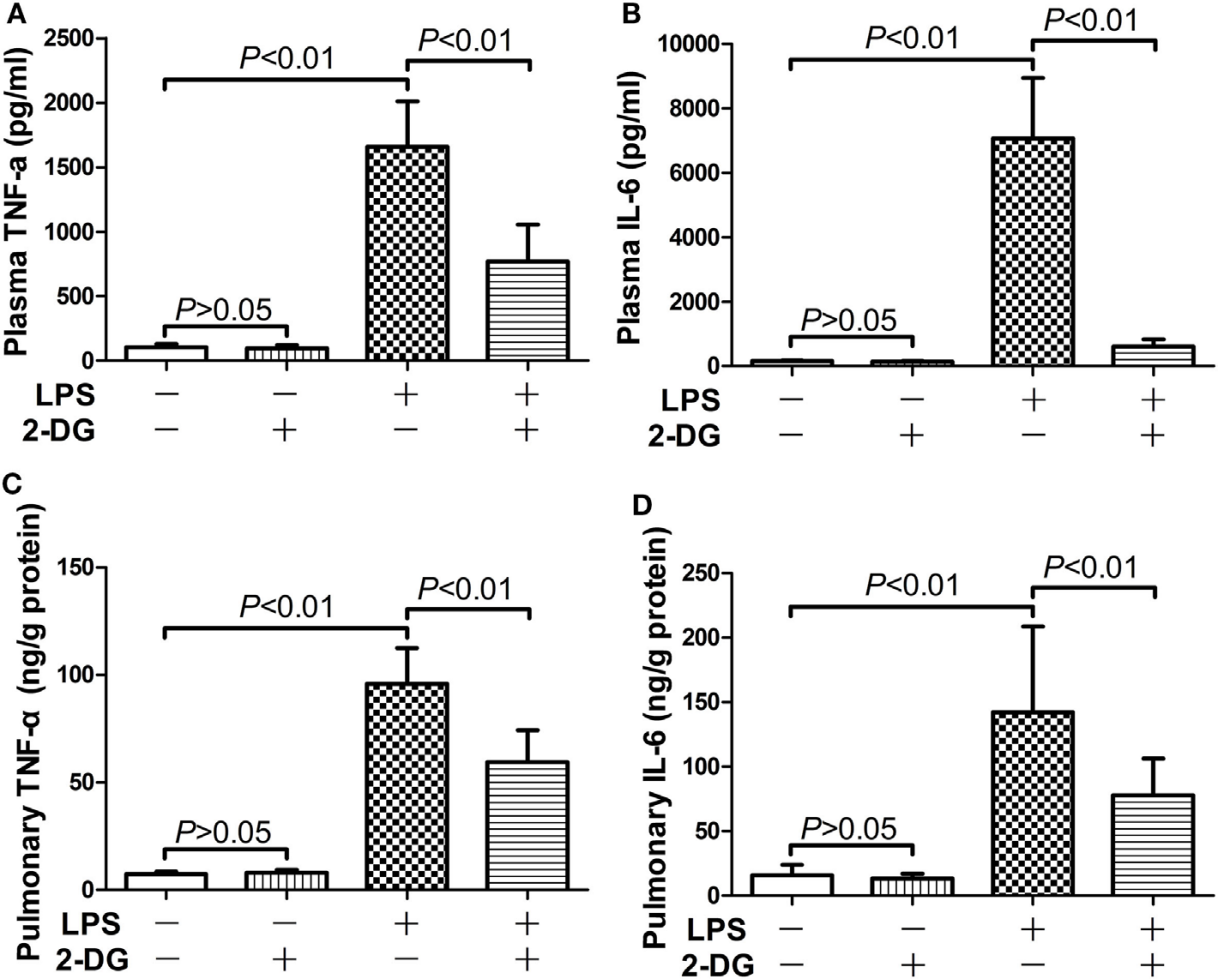
2-Deoxyglucose (2-DG) mitigated lipopolysaccharide (LPS)-induced production of pro-inflammatory cytokines. Mice with LPS-induced lethal inflammation were treated with vehicle or 2-DG 30 min before LPS exposure. The plasma samples and lung samples were harvested 18 h after LPS exposure. The plasma level of **(A)** tumor necrosis factor alpha (TNF-α) and **(B)** interleukin 6 (IL-6) as well as the pulmonary level of **(C)** TNF-α and **(D)** IL-6 were determined by enzyme-linked immunosorbent assay. Data were expressed as mean ± SD, *n* = 8.

The potential anti-inflammatory effects of 2-DG were investigated in mice with lethal endotoxemia. The results showed that treatment with 2-DG significantly alleviated LPS-induced histological abnormalities, including pulmonary edema and leukocytes infiltration (Figures 1A,B). The evaluation of Evans blue leakage and the determination of MPO activities also indicated that LPS-induced pulmonary edema and leukocytes infiltration were attenuated by 2-DG (Figures 1C,D). In the survival analysis (Figure 1E), more than 90% of LPS-exposed mice died within 72 h, but the mortality rate dropped to 15% in 2-DG-treated group. We next investigated whether the production of pro-inflammatory cytokines could be modulated by 2-DG. The data showed that pretreatment with 2-DG resulted in reduced induction of TNF-α (Figure 2A) and IL-6 (Figure 2B). Consistently, LPS-induced elevation of pulmonary TNF-α (Figure 2C) and IL-6 (Figure 2D) were also suppressed by 2-DG. These data indicate that the treatment with 2-DG might result in beneficial outcomes in LPS-induced lethal inflammation.

### 2-DG Suppressed LPS-Induced Upregulation of Nuclear PKM2

**Figure 3:**
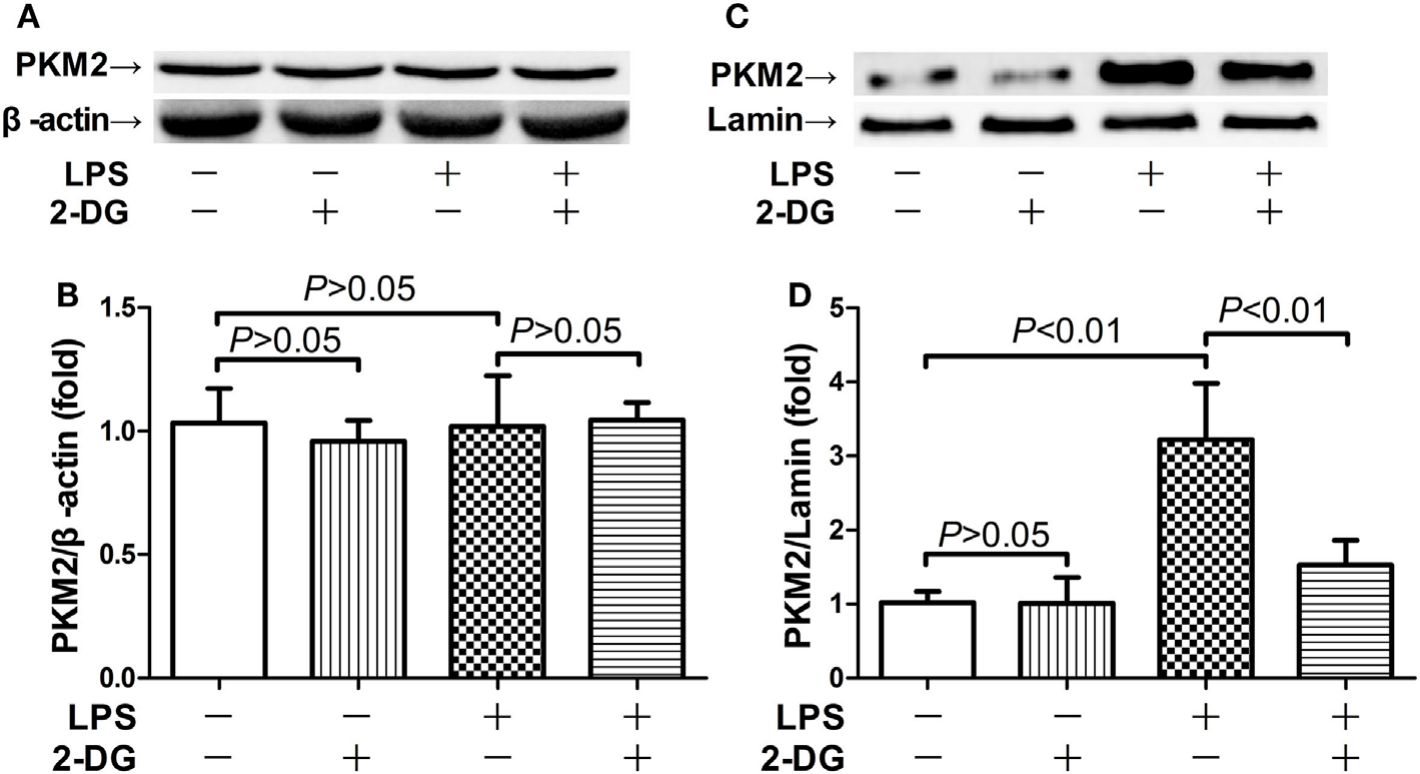
2-Deoxyglucose (2-DG) suppressed lipopolysaccharide (LPS)-induced nuclear accumulation of pyruvate kinase M2 (PKM2). Mice with LPS-induced lethal inflammation were treated with vehicle or 2-DG 30 min before LPS exposure. The lung samples were harvested 18 h after LPS administration, and the level of total PKM2 **(A,B)** and nuclear PKM2 **(C,D)** was detected by western blot analysis. Data were expressed as mean ± SD, *n* = 4.

Because nuclear PKM2 is crucial for the bridge between metabolism and inflammation, the level of total PKM2 in pulmonary homogenates and the level of nuclear PKM2 in nuclear extracts have been determined. The data indicate that the total PKM2 level remained unchanged after LPS exposure and/or 2-DG treatment (Figures 3A,B). However, LPS induced significant elevation of PKM2 level in the nuclei, but the treatment with 2-DG reduced the nuclear level of PKM2 in LPS-exposed mice (Figures 3C,D). These data suggest that the treatment with 2-DG might suppress LPS-induced upregulation of nuclear PKM2 in the lung tissue.

### Inhibition of Nuclear Accumulation of PKM2 Attenuated Inflammatory Injury

**Figure 4:**
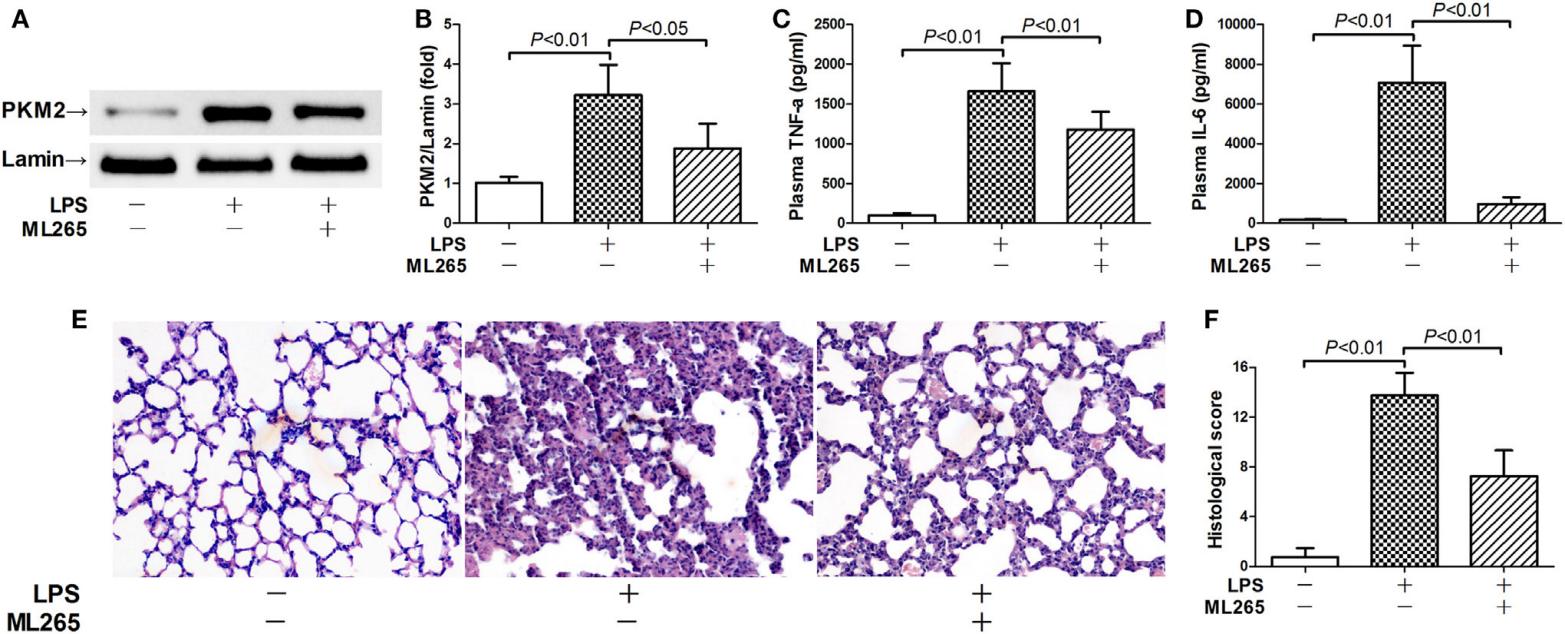
Prevention of pyruvate kinase M2 (PKM2) nuclear accumulation attenuated lipopolysaccharide (LPS)-induced inflammatory injury. Mice with LPS-induced lethal inflammation were treated with ML265 30 min before LPS exposure. The lung samples and plasma samples were harvested 18 h after LPS exposure. **(A,B)** The level of nuclear PKM2 was determined by western blot analysis. Data were expressed as mean ± SD, *n* = 4. The plasma levels of **(C)** tumor necrosis factor alpha (TNF-α) and **(D)** interleukin 6 (IL-6) were determined by enzyme-linked immunosorbent assay (ELISA). Data were expressed as mean ± SD, *n* = 8. **(E)** The lung sections were stained with hematoxylin and eosin for morphological evaluation, and the representative lung sections of each group were shown (original magnification: 400×). **(F)** The histopathological abnormalities of the lung sections were blindly scored.

In the next step, the potential significance of nuclear PKM2 in LPS-induced inflammatory injury was investigated by pharmacological prevention of PKM2 nuclear accumulation with ML265. ML265 is a small molecule that binds with PKM2 and increases the metabolic activity of PKM2 in the cytoplasm but prevents its nuclear translocation (15). Our data showed that ML265 decreased the nuclear level of PKM2 in LPS-challenged mice (Figures 4A,B). This alteration was associated with suppressed induction of TNF-α and IL-6 (Figures 4C,D). In addition, the treatment with ML265 also alleviated the LPS-induced histological abnormalities in the lung tissue (Figures 4E,F). These data suggest that nuclear PKM2 might play important roles in LPS-induced inflammatory injury in the lung.

### STAT3 Contributed to the Pro-inflammatory Activities of Nuclear PKM2

**Figure 5:**
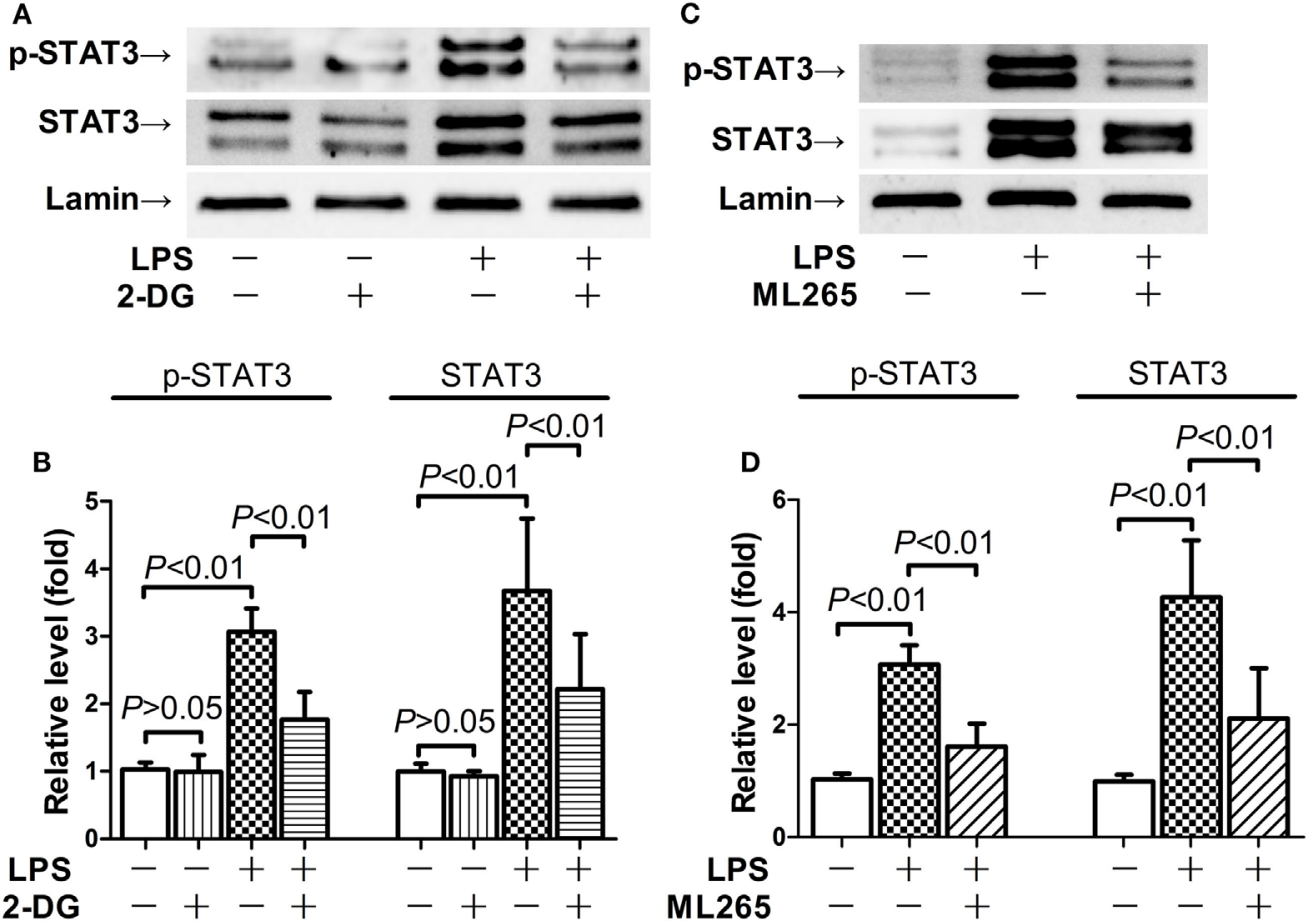
2-Deoxyglucose (2-DG) and ML265 suppressed lipopolysaccharide (LPS)-induced phosphorylation of nuclear signal transducer and activator of transcription 3 (STAT3). **(A,B)**. Mice with LPS-induced lethal inflammation were treated with 2-DG 30 min before LPS exposure. The lung samples were harvested 18 h after LPS exposure, and the level of phosphorylated STAT3 and total STAT3 in the nuclear extracts was determined by western blot analysis. **(C,D)** Mice with LPS-induced lethal inflammation were treated with vehicle or ML265 30 min before LPS exposure. The lung samples were harvested 18 h after LPS exposure, and the level of phosphorylated STAT3 and total STAT3 in the nuclear extracts was determined by western blot analysis. Data were expressed as mean ± SD, *n* = 4.

**Figure 6:**
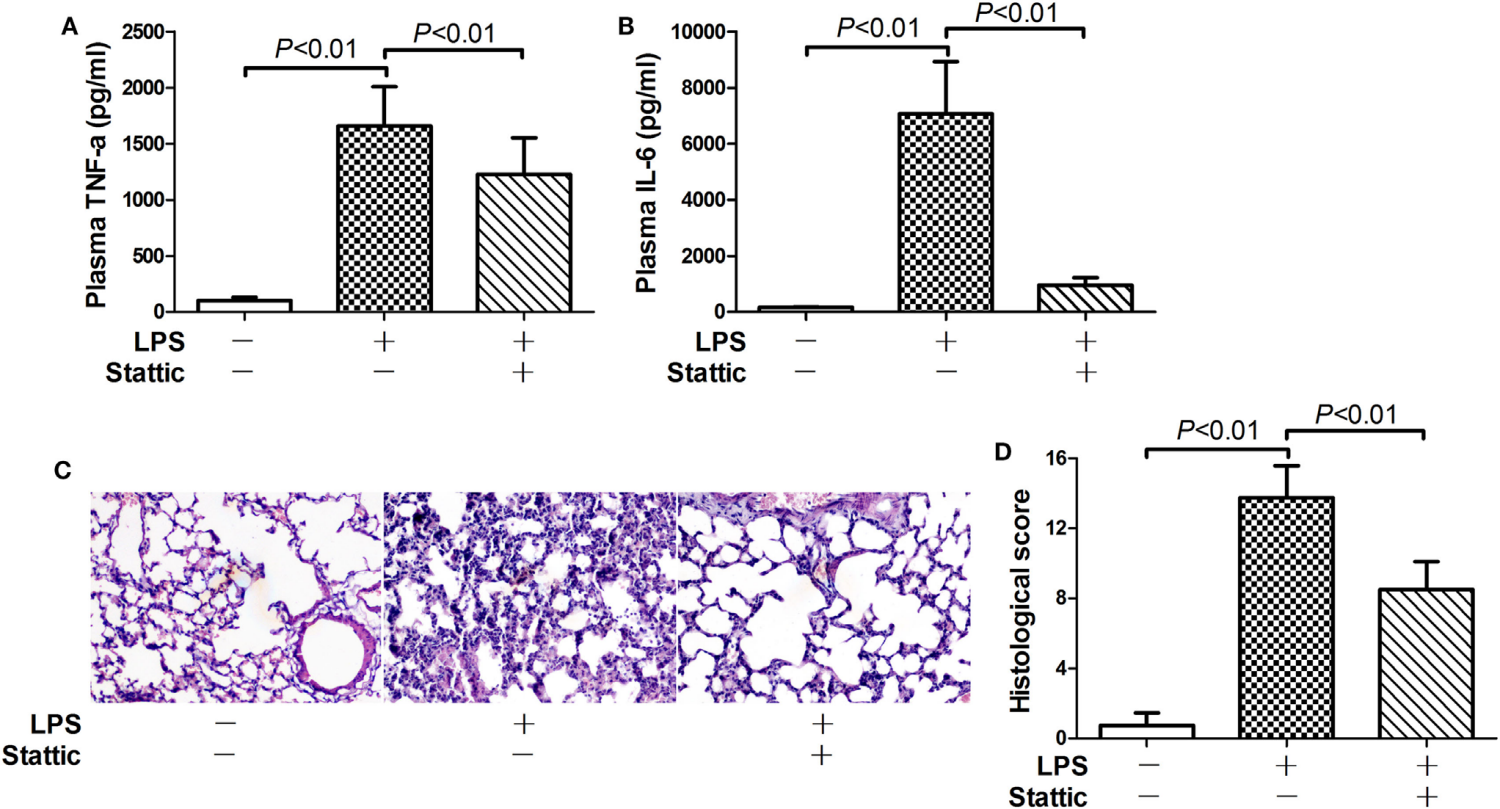
Inhibition of signal transducer and activator of transcription 3 alleviate lipopolysaccharide (LPS)-induced inflammatory injury. Mice with LPS-induced lethal inflammation were treated with stattic 30 min before LPS exposure. The plasma samples and lung samples were harvested 18 h after LPS exposure. The plasma level of **(A)** tumor necrosis factor alpha (TNF-α) and **(B)** interleukin 6 (IL-6) were determined by enzyme-linked immunosorbent assay. Data were expressed as mean ± SD, *n* = 8. **(C)** The lung sections were stained with hematoxylin and eosin for morphological evaluation, and the representative lung sections of each group were shown (original magnification: 400×). **(D)** The histopathological abnormalities of the lung sections were blindly scored.

Nuclear PKM2 might act as a protein kinase that phosphorylates inflammation-related transcription factors such as STAT3 (15, 17). We then questioned whether STAT3 is involved in the pro-inflammatory activities of nuclear PKM2. The results indicate that the level of total STAT3 remained unchanged in mice after LPS exposure and/or 2-DG treatment (Figure S1 in Supplementary Material). However, treatment with 2-DG suppressed the phosphorylation of nuclear STAT3 and reduced the level of nuclear STAT3 (Figures 5A,B). In addition, inhibition of PKM2 nuclear accumulation by ML265 also resulted in decreased phosphorylation and reduced the level of STAT3 in the nuclear extracts (Figures 5C,D). To investigate the roles of STAT3 in LPS-induced lethal inflammation, a STAT3 inhibitor stattic was administered in the experimental animals. The results indicate that treatment with stattic significantly decreased the plasma level of TNF-α and IL-6 (Figures 6A,B). Meanwhile, LPS-induced histological lesions in the lung tissue was also mitigated by stattic (Figures 6C,D). These data suggest that the phosphorylation and activation of nuclear STAT3 might be involved in the pro-inflammatory properties of nuclear PKM2, and the anti-inflammatory benefits of 2-DG might be associated with suppressed PKM2-STAT3 signaling in the nuclei.

### Posttreatment with 2-DG Improved the Survival of LPS-Insulted Mice

**Figure 7:**
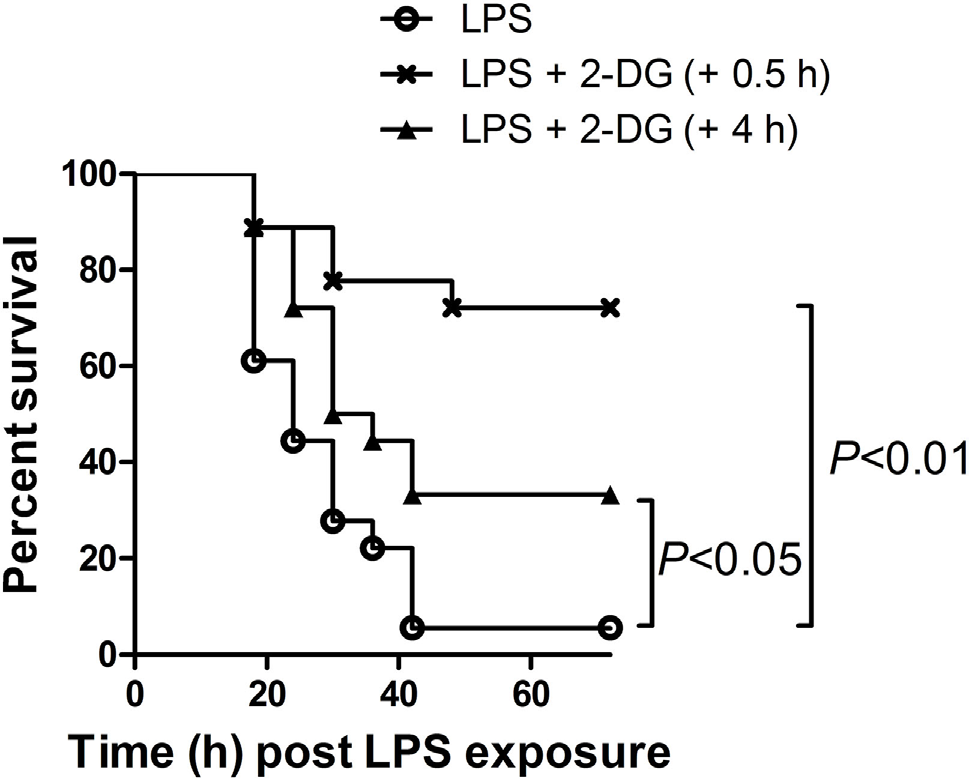
Posttreatment with 2-deoxyglucose (2-DG) improved the survival of lipopolysaccharide (LPS)-insulted mice. Mice with LPS-induced lethal inflammation were treated with 2-DG 0.5 or 4 h post-LPS exposure, the mortality of the animals was monitored, and the percent survival rate was expressed as a Kaplan–Meier survival curves, *n* = 18.

Since preinsult treatment protocol is impractical under most clinical situations, this study also tested the potential benefits of 2-DG treatment post-LPS exposure. Interestingly, the survival analysis showed that treatment with 2-DG 0.5 h post-LPS exposure significantly increased the survival rate of the experimental animals (Figure 7). In addition, treatment with 2-DG 4 h post-LPS exposure also resulted in beneficial outcomes (Figure 7). However, treatment with 2-DG 12 or 24 h post-LPS exposure has no obvious effects on the survival rate of LPS-exposed mice (Figure S2 in Supplementary Material).

## Discussion

Inflammation is a highly reactive response, which requires intensive metabolic support (23). Restriction of nutrition supply/metabolism by CR or pharmacological approaches has significant suppressive effects on inflammatory injury (6). In this study, treatment with 2-DG, a representative CRM, provided anti-inflammatory benefits in mice with lethal dose of LPS-induced inflammatory injury as evidenced by suppressed production of pro-inflammatory cytokines, alleviated lung injury, and improved survival rate. These data suggest that the treatment with 2-DG might become a novel approach to protect against lethal inflammation.

Inflammation is usually associated with marked metabolic reprogramming, and PKM2 plays crucial roles in driving these metabolic alterations and promoting inflammatory response (15). It was reported that prevention of PKM2 nuclear translocation alleviated LPS-induced production of pro-inflammatory cytokines (24, 25). This study also found that nuclear PKM2 played crucial roles in LPS-induced inflammatory injury because prevention of nuclear accumulation of PKM2 by ML265 significantly alleviated lung injury. In addition, we found that the anti-inflammatory benefits of 2-DG were associated with the reduced level of nuclear PKM2, suggesting that treatment with 2-DG might interrupt the association between metabolism and inflammation *via* decreasing the level of nuclear PKM2.

It has been suggested that nuclear translocation of PKM2 is a critical molecular event in inflammatory response (15). However, the mechanism through which inflammatory stimuli induce PKM2 translocation remains largely unknown. A recent study has suggested that PKM2 is a redox-sensitive molecule, and the nuclear translocation of PKM2 could be modulated by oxidation and 2-DG suppressed PKM2 nuclear accumulation *via* regulating oxidative stress (19). In addition, posttranslational modifications such as phosphorylation, acetylation, hydroxylation, and SUMOylation are also involved in the regulation of PKM2 nuclear accumulation (26), but the detailed mechanisms mediating these modifications under inflammatory conditions remain unknown.

Signal transducer and activator of transcription 3 is a pivotal regulator with profound regulatory activities in inflammatory response (27). Although conditional deletion of STAT3 in macrophages, neutrophils, or endothelial cells resulted in exacerbated inflammatory injury (28, 29), genetic reduction of STAT3 or pharmacological inhibition of STAT3 was associated with suppressed inflammation both *in vitro* and *in vivo* (30, 31). Therefore, STAT3 inhibition in this study might attribute to the anti-inflammatory benefits of 2-DG. Similarly, STAT3 inhibition was suggested to be responsible for the anti-inflammatory effects of rapamycin, IL-35, and high-density lipoprotein (32–34).

Signal transducer and activator of transcription 3 has been confirmed as a major downstream target of nuclear PKM2 (19). The nuclear PKM2 functions as a protein kinase that directly phosphorylates STAT3 at Tyr^705^(17). The phosphorylation of STAT3 at Tyr^705^ has been regarded as a critical modification for the pro-inflammatory effects of STAT3 (35, 36). Several studies have found that STAT3 activation was required for the inflammation regulatory effects of nuclear PKM2 (18, 19). Therefore, 2-DG-induced STAT3 inhibition might result from the reduced level of nuclear PKM2. Recent studies also found that treatment with 2-DG abrogated STAT3 activation in leukemic cells and fibrosarcoma cells, but the underlying mechanisms were unclear (37, 38). Because nuclear accumulation of PKM2 is suggested to be involved in the progression of various tumor (39, 40), it is a worthy concern that whether inhibition of PKM2 nuclear accumulation is responsible for the suppressed activation of STAT3 by 2-DG in tumor cells.

Because preinsult administration of 2-DG is unpractical in clinical situation, we also tested the pharmacological significance of posttreatment with 2-DG. Interestingly, we found that treatment with 2-DG at the early stage post-LPS exposure could also improve the survival rate. However, treatment with 2-DG at the late stage post-LPS exposure failed to improve the survival rate. These data suggest that the CRM 2-DG might have potential value for the early intervention of inflammatory injury.

It has been reported recently that PKM2 is expressed or functions in several inflammation-related cells such as macrophages, neutrophils, and endothelial cells (41–44). All of these cells are crucial players in inflammatory response and acute lung injury (45). In addition, LPS exposure increased the nuclear level of phosphorylated STAT3 in the resident cells, such as endothelial cells and smooth muscle cells, as well as in the recruited inflammatory cells such as monocytes and neutrophils (46). Investigation of the pathological significance of the nuclear PKM2-STAT3 pathway in certain cells might be very interesting. However, the pharmacological interventions in this study could not provide the experimental data in certain cell types. The detailed mechanisms underlying the nuclear PKM2-STAT3 pathway in inflammatory disorders require more intensive studies.

Taken together, this study found that treatment with 2-DG, a representative CRM, provide significant anti-inflammatory benefits in mice with LPS-induced lethal inflammation. These effects might be associated with suppressed nuclear accumulation of PKM2 and reduced phosphorylation of STAT3. Most interestingly, posttreatment with 2-DG at the early stage also resulted in beneficial outcomes. Therefore, 2-DG might have potential value in the early intervention of lethal inflammatory injury.

## Ethics Statement

This study was carried out in accordance with the recommendations of the Animal Care and Use Committee of Chongqing Medical University. The protocol was approved by the Animal Care and Use Committee of Chongqing Medical University.

## Author Contributions

KH and LZ planned the experiments. KH, KF, and RJ performed the experiments. LL and QA analyzed the data. JD prepared the figures. YY and PG drafted the manuscript. JW and LZ proof read the final version of the manuscript.

## Conflict of Interest Statement

The authors declare that the research was conducted in the absence of any commercial or financial relationships that could be construed as a potential conflict of interest.
