# Supplementary material for: Caloric Restriction Mimetic 2-Deoxyglucose Alleviated Inflammatory Lung Injury via Suppressing Nuclear Pyruvate Kinase M2–Signal Transducer and Activator of Transcription 3 Pathway
Source: Front Immunol. 2018 Mar 2;9:426. doi: 10.3389/fimmu.2018.00426 (PMC5840172; doi:10.3389/fimmu.2018.00426)
Supplement: Supplementary file 1 [file presentation_1.PDF]

Supplementary Information

Supplementary Figure 1. 2-DG have no obvious effects on the total level of STAT3 in lung tissue. Mice with LPS-induced lethal inflammation were treated with vehicle or 2-DG 30 mim before LPS exposure. The lung samples were harvested 18 h after LPS administration, the total level of STAT3 in lung tissue was detected by western blot analysis. Data were expressed as mean ± SD, n=4.

Supplementary Figure 2. Post-treatment with 2DG at late stage post LPS exposure failed to improve the survival of the experimental animals. Mice with LPS-induced lethal inflammation were treated with 2-DG 12 or 24 h post LPS exposure, the mortality of the animals was monitored and the percent survival rate was expressed as a Kaplan-Meier survival curves, n=18.
