# Supplementary figures and images for: Caloric Restriction Mimetic 2-Deoxyglucose Alleviated Inflammatory Lung Injury via Suppressing Nuclear Pyruvate Kinase M2–Signal Transducer and Activator of Transcription 3 Pathway
Source: Front Immunol. 2018 Mar 2;9:426. doi: 10.3389/fimmu.2018.00426 (PMC5840172; doi:10.3389/fimmu.2018.00426)

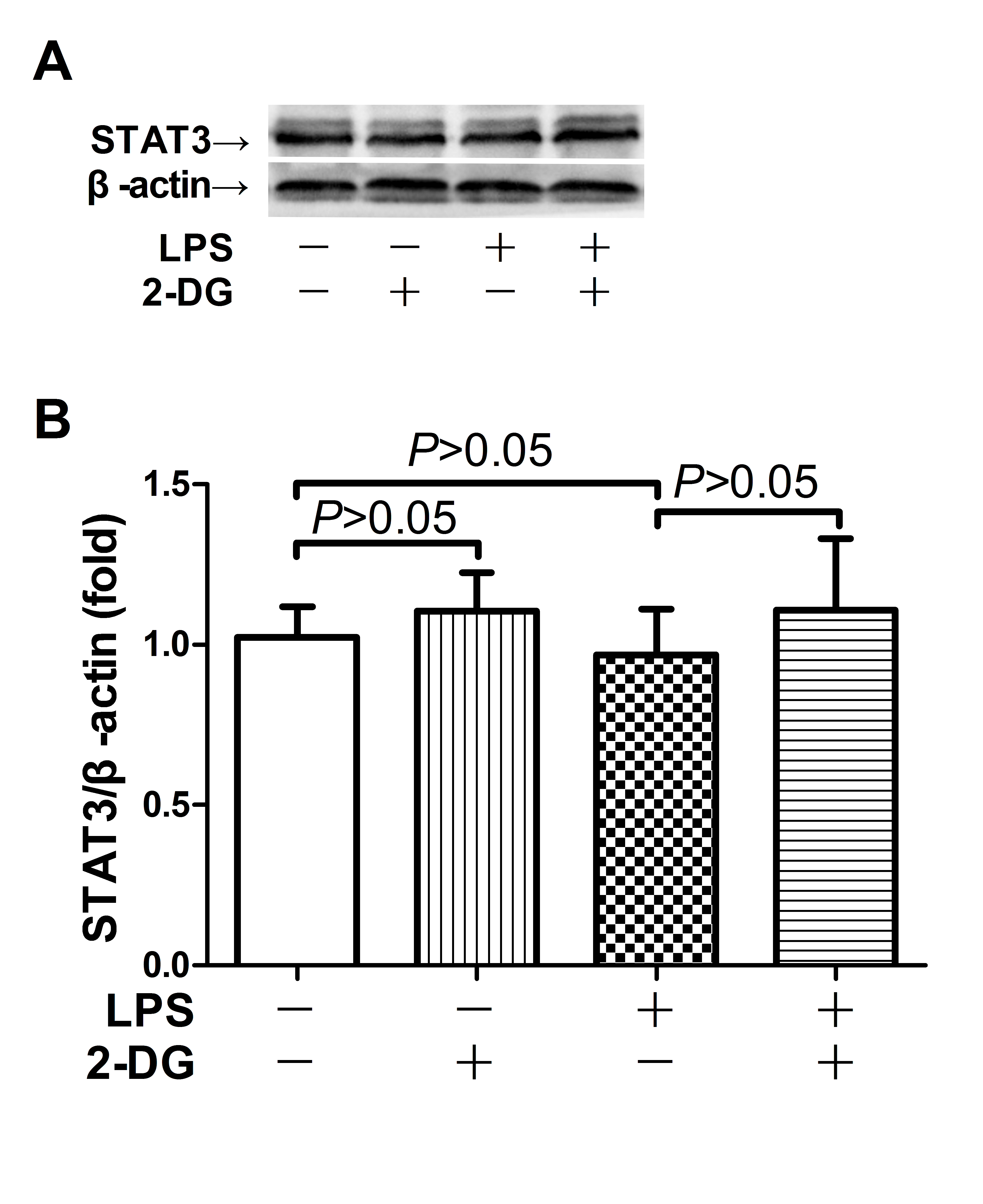

Supplement: Supplementary file 2 [file image_1.TIF]

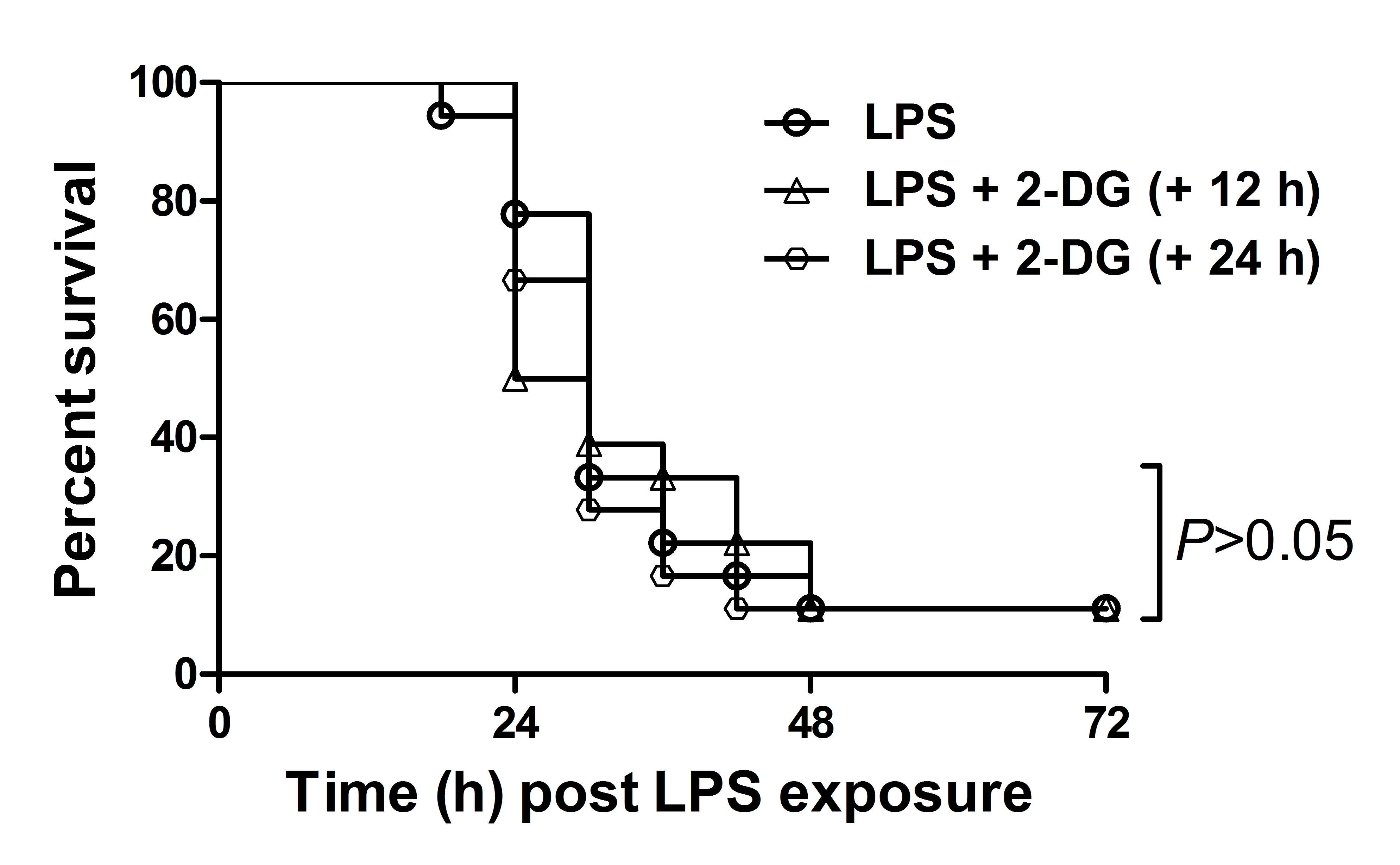

Supplement: Supplementary file 3 [file image_2.TIF]
